# Physiological and Disease Models of Respiratory System Based on Organ-on-a-Chip Technology

**DOI:** 10.3390/mi12091106

**Published:** 2021-09-15

**Authors:** Di Wang, Ye Cong, Quanfeng Deng, Xiahe Han, Suonan Zhang, Li Zhao, Yong Luo, Xiuli Zhang

**Affiliations:** 1 College of Pharmaceutical Science, Soochow University, Suzhou 215123, China; diwang0028@163.com (D.W.); 20204026006@stu.suda.edu.cn (Q.D.); 20194226017@stu.suda.edu.cn (X.H.); 2 Department of Pulmonary and Critical Care Medicine, Second Medical School, China Medical University, Shenyang 110004, China; 3 State Key Laboratory of Fine Chemicals, Department of Pharmaceutical Sciences, School of Chemical Engineering, Dalian University of Technology, Dalian 116023, China; cyciel96@163.com (Y.C.); zhangsuonan@mail.dlut.edu.com (S.Z.)

**Keywords:** organ-on-a-chip, microfluidic chip technology, respiratory diseases, cell co-culture

## Abstract

The pathogenesis of respiratory diseases is complex, and its occurrence and development also involve a series of pathological processes. The present research methods are have difficulty simulating the natural developing state of the disease in the body, and the results cannot reflect the real growth state and function in vivo. The development of microfluidic chip technology provides a technical platform for better research on respiratory diseases. The size of its microchannel can be similar to the space for cell growth in vivo. In addition, organ-on-a-chip can achieve long-term co-cultivation of multiple cells and produce precisely controllable fluid shear force, periodically changing mechanical force, and perfusate with varying solute concentration gradient. To sum up, the chip can be used to analyze the specific pathophysiological changes of organs meticulously, and it is widely used in scientific research on respiratory diseases. The focus of this review is to describe and discuss current studies of artificial respiratory systems based on organ-on-a-chip technology and to summarize their applications in the real world.

## 1. Introduction

**Figure 1:**
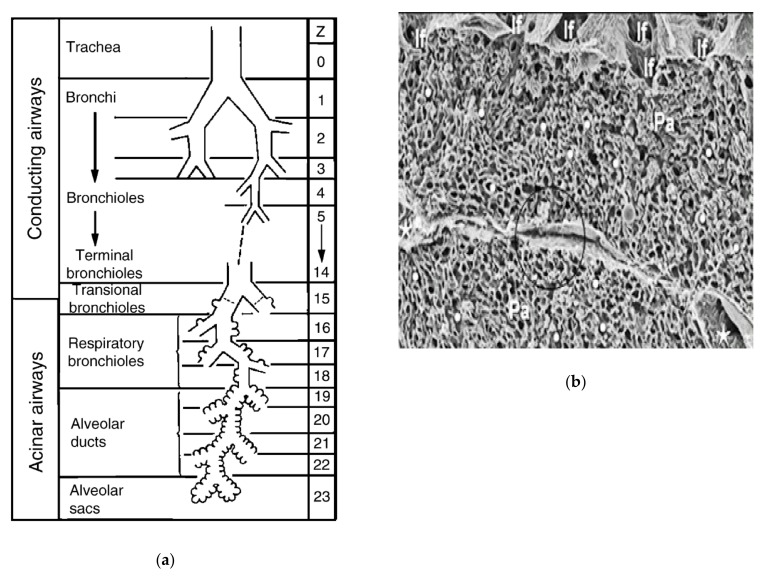
Lung structure and internal ventilation structure: (**a**) Schematic of the branching airways in Weibel’s model; (**b**) a scanning electron micrograph from a human lung that shows the capillary network in 3D. Reproduced with permission from [1,3].

The respiratory system is one of the most complex systems and an indispensable organ for human survival. The lung is the most important functional organ in the entire respiratory system, and it is mainly divided into two parts: the conducting airways and the gas exchange portions. The conducting airways, which are primarily for the role of transmitting gas, are composed of 16 generations of bronchi, and the structure of each generation is also very complex (Figure 1a) [1], while the gas exchange portions are composed of respiratory bronchioles and alveolar ducts and sacs, which contract and relax with breathing, mainly play the role of gas exchange (Figure 1b). The two parts cooperate to keep breathing normally [2].

The occurrence and development of respiratory system diseases often involve a series of pathological processes in both two parts. For example, in chronic obstructive pulmonary disease (COPD), characteristic pathological changes exist in the central airway, peripheral airway, lung parenchyma, and vascular systems. In the central airway, mucus secretion increases, and inflammation infiltrates the mucosal epithelium, causing the mucus glands and goblet cells to increase [4]. In the peripheral airway, repeated airway damaged and repaired processes are caused by chronic inflammation, resulting in airway wall remodeling and narrowing, leading to fixed airway obstruction. Excessive swelling of alveoli, decreased elasticity, and increased emphysema can be seen in the lung parenchyma, while the pulmonary vessel wall is significantly thickened [5]. Due to changes in lifestyle and environmental factors, the incidence of respiratory system diseases is increasing yearly, becoming one of the global problems that need to be solved urgently. Pneumonia and COPD are the main causes of death within respiratory diseases. According to the World Health Organization, in 2017, pneumonia caused an estimated 808,694 deaths of children under five years of age, accounting for 15% [6]. In 2015, an estimated 3.17 million people died of COPD worldwide, equivalent to 5% of all deaths worldwide in the same year [7]. In addition, lung cancer, acute respiratory distress syndrome (ARDS), idiopathic pulmonary fibrosis (IPF), and other diseases also have high mortality rates in fatal diseases.

However, in general, the cognition of respiratory diseases is not deep enough, and many key molecules in the pathological process need to be further verified. On the one hand, it is due to the complicated pathogenesis of respiratory diseases, and on the other hand, it is difficult to simulate the real state of the diseases in the body with the existing research methods; the results do not reflect the actual state of cell growth.

**Figure 2:**
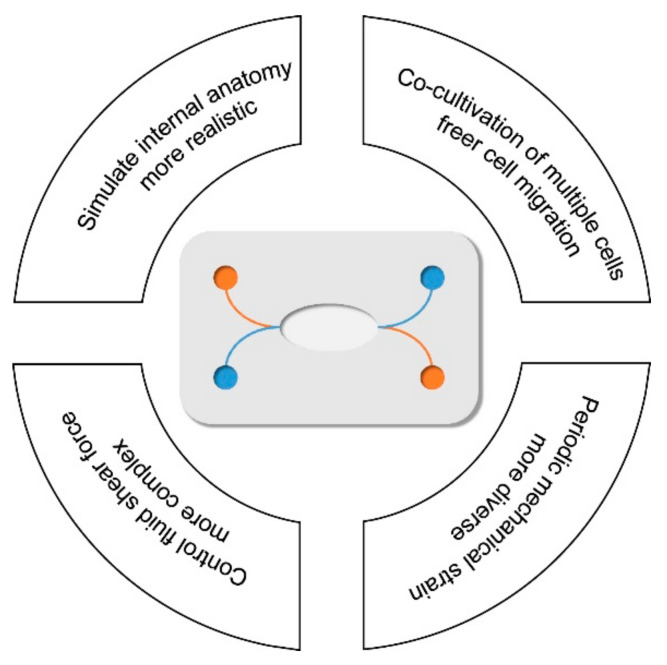
Four main advantages of microfluidic technology.

In recent years, microfluidic technology has provided a better technical platform for research on human diseases. Its main advantages are summarized in Figure 2. The similarity of its microchannel spatial dimensions and the cell growth in vivo is incredible, and complicated multidimensional structures may be formed to support study interactions between cells. Cells can freely stretch, polarize, migrate, and cluster in these structures, closer to the real situation in the body [8]. In addition, the materials of chips are suitable for cell culture due to good light permeability and high biocompatibility and can continuously supply fresh nutrients and oxygen to the cells through an external pump, so they can better simulate the survival of cells in vivo. Moreover, they can also generate precise and controllable fluid shear force, periodically changing mechanical strain and perfusate with solute concentration gradient changes [9], which are difficult to achieve in traditional cell culture models. Based on this, the microfluidic technology can be used for detailed analysis of the tissue- and organ-specific stress responses, such as the response to drugs, toxins, recruitment of circulating immune cells, or other stimulating factors.

With the development of scientific research, microfluidic chip technology has gradually expanded from cells to tissues and organs. Because it can achieve long-term co-cultivation of multiple cells, it has outstanding advantages in the simulation of the tissue-tissue contact interface and the organ microenvironment, such as the tissue interface of vascular endothelial cells, surrounding tissues, and parenchymal cells, which is indispensable to immunity and humoral regulation in the body [10,11]. Additionally, multiple chips can be connected by fluid or blood endothelial tissue in accordance with the relationship in vivo. It is possible to simulate the physiological interactions and drug distribution of different organs and tissues throughout the whole body [12,13].

The anatomical structure and physiological functions of lung tissue are complex. Both air ducts and respiratory parts are involved in many complex diseases, including many tissues and cell–cell interactions. The organ chip is suitable for simulating the micro-environment where alveolar epithelial cells are located, especially the “air–blood barrier”. In 2010, Huh and others of Harvard University reconstituted the critical functional alveolar–capillary interface of the human lung using microfluid technology. This model created an unprecedented lung-on-a-chip in which the response of the alveoli to bacteria and inflammatory cytokines was mainly studied [14]. Subsequently, lung-on-a-chip technology used in an increasing number of studies has shown great potential in studying human lung physiology and respiratory diseases. With the continuous innovation of technology, organ-on-a-chip for the respiratory system has become more and more specialized in recent years to deal with various complex diseases. Most of the emerging reviews are for a specific disease or field; however, few articles have systematically explained the research progress of microfluidic technology in the respiratory system. This review systematically classifies and comprehensively summarizes the latest research progress of physiology and disease models in lung-on-a-chip so as to provide more valuable and easier-to-find information for subsequent researchers.

## 2. Physiological Model of Respiratory System Based on Organ-on-a-Chip

**Figure 3:**
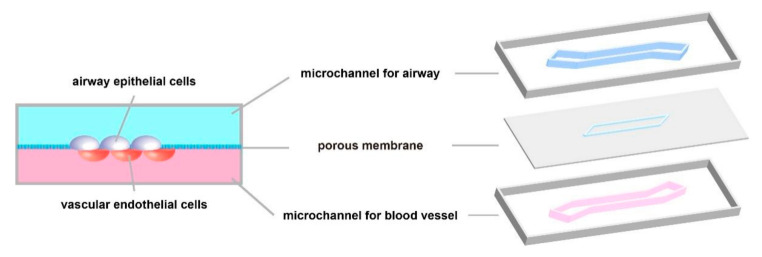
Common dual-channel model of cell co-culture. The upper channel is exposed to the air for culturing epithelial cells; the culture fluid flows in the lower channel for culturing vascular endothelial cells.

The respiratory system can be seen as a large tree-like structure, of which physiological structure and functions are complicated. In short, the airway and alveolar surfaces are covered with epithelial cells, which vary in type and composition at different branching stages. Because of this, the conducting airways and gas exchange portions of the respiratory system can perform different physiological functions [15]. In addition, there are capillaries around the airways and alveoli, which provide a steady stream of nutrients for the survival of epithelial cells. With the development of lung-on-a-chip, it has become possible to integrate airway epithelial cells and vascular endothelial cells into the same in vitro model. Therefore, the existing organs-on-a-chip of the physiological respiratory system mainly focus on the dual-chamber channel model for culturing two kinds of cells mentioned above (Figure 3). The model successfully simulated the microenvironment of the alveolar–capillary barrier and studied the communication between various epithelial cells. In addition, it is also possible to simulate the real pulmonary anatomical structure with respiratory function by changing the geometry of the micro-channel or the perfusion material, which provides a solid basis for studying the various physiological processes of the respiratory system in vitro. This section elaborates on the application of lung-on-a-chip in the above aspects.

### 2.1. In Vitro Alveolar–Capillary Barrier Models Using Organ-on-a-Chip

The most important physiological function of the lung is respiration, which mainly occurs at the alveolar–capillary barrier through gas exchange between oxygen and carbon dioxide at the distal end of the alveoli and the pulmonary capillaries. In the body, about 600 million alveoli with a diameter of 200 μm are packed tightly into a spongy structure that interweaves with the pulmonary capillary to form a nearly 70 square meter surface for gas exchange [16]. The alveolar–capillary barrier, also known as the alveolar lining layer (ALL), is composed of alveolar surfactant [17], alveolar epithelial cells, an extracellular matrix, vascular endothelial cells, plasma, and erythrocyte membranes [18,19].

**Figure 4:**
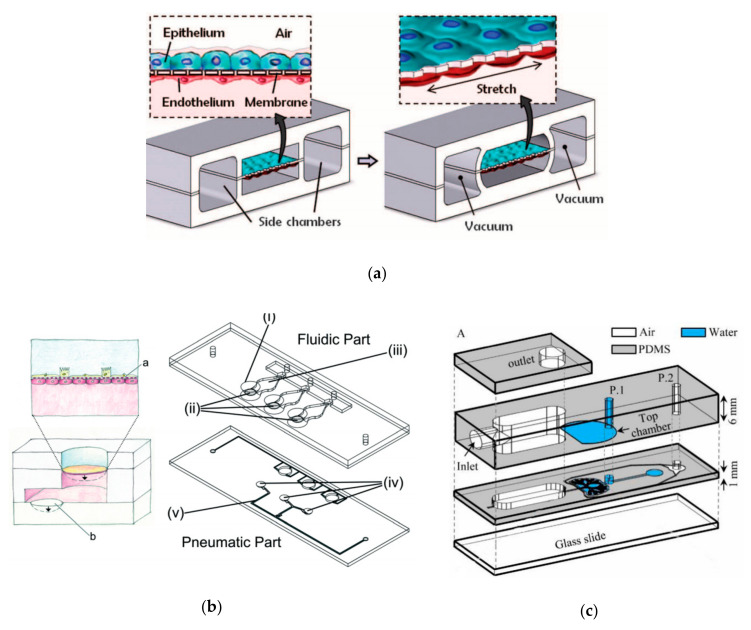

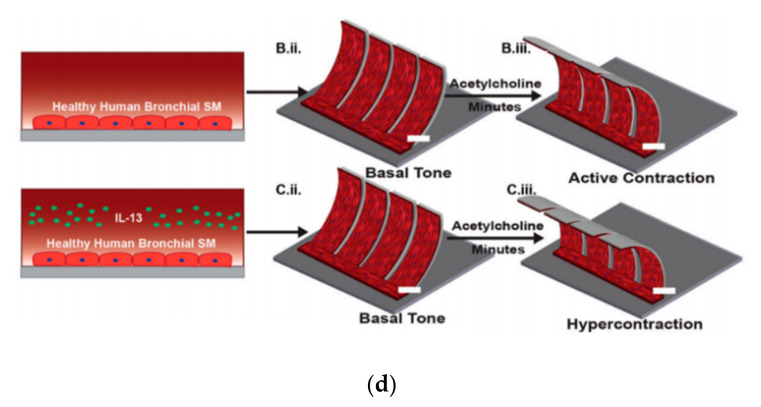
Several representative physiological models of respiratory system based on lung-on-a-chip: (**a**) the lung-on-a-chip that simulates the breathing process through air pressure changes in the side channel produced by Huh et al.; (**b**) the lung-on-a-chip that simulates the breathing process through basement membrane deformation produced by Stucki et al.; (**c**) the lung-on-a-chip that can directly observe the position of particles produced by Fishler et al.; (**d**) the lung-on-a-chip with a special structure that simulates the contraction and relaxation of bronchial smooth muscle produced by Nesmith et al.. Reproduced with permission from [14,40,53,57].

In 2004, Hermanns et al. established a co-culture system of human epithelial cells and microvascular endothelial cells through the NCI H441 and HPMEC cell lines. Under the stimulation of dexamethasone, the contact inhibition of differentiated monolayer cells was successfully achieved, and the typical junction structure of polarized monolayer epithelial cells was observed under an electron microscope. In addition, this study measured transepithelial electrical resistance (TEER) in a co-culture system for the first time, which proved that the epithelial tissue grew well in this model. TEER has been an essential monitor for evaluating the growth of epithelial tissue in the subsequent lung in vitro model studies [20]. This model provides a basis for investigating the interaction between epithelium and endothelium in lung diseases [11]. Subsequently, Huh et al. produced a lung-on-a-chip capable of breathing freely (Figure 4a). The chip contains three parallel microfluidic channels, and the central channel is divided into two levels, which are separated by porous membranes. Alveolar epithelial cells and pulmonary microvascular endothelial cells were cultured on both sides of the membrane, respectively, simulating the gas phase and the blood phase, successfully realizing the simulation of the alveolar–capillary barrier. Alveolar epithelial cells and microvascular endothelial cells are attached to the opposite surface of the porous membrane to form a complete monolayer containing epithelium and endothelial connexin, occludin, and vascular endothelial cadherin (VE-cadherin). These cells can be kept moist with the help of surfactants and survive for about two weeks. The separation of the porous membrane not only helps to deliver nutrients from the blood phase to the surface of epithelial cells, but also allows a pressure difference between the middle channel and the channels on both sides. During the initiation of breathing, the pressure in the pleural cavity decreases, causing the alveoli to expand. When air is breathed in, the alveolar–capillary interface is stretched. In this model, when the side channel is connected to the vacuum, the internal pressure can be reduced, causing the expansion of the middle channel and the stretching of alveolar–capillary barrier on the porous membrane. This process simulates changes in the mechanical strain during breathing in vivo. Adding inflammatory factor TNF-a or increasing bacterial exposure was used to simulate the inflammatory response in the model. Significant upregulation of ICAM1 was detected, and the recruitment and migration of neutrophils from the microvascular lumen to alveoli are significantly enhanced [14]. Huh et al. realized the co-cultivation of multiple types of cells at the air–liquid interface, and the mechanical strain generated by respiration is considered in the model. In addition, it also introduces the trans-barrier transport of pathogens and cells. Today, it is still a leading test platform in the field of lung-on-a-chip.

Based on this model, other simpler platforms that can produce stable results have been produced, realizing the simulation of functional alveolar units. To put it simply, the simplified platforms consist of dual-chambered microchannels. The upper channel is exposed to air, while the lower channel is filled with culture medium or human blood. The dual microchannels were separated by a porous polydimethylsiloxane (PDMS) membrane on which culture epithelial cells (alveolar epithelial cells or bronchial epithelial cells) and endothelial cells achieve a simple ALL barrier [21]. This model provides good support for the treatment of diseases, such as the simulation of pulmonary edema [22], development of lung cancer [23], pulmonary thrombosis [24], and COPD [25]. We will elaborate on it in the next section in detail. The model is similar to the chip design of Huh, but no mechanical strain is added to the model, making it easier and more repeatable. Therefore, it is widely used to study some pathophysiological processes that are not related to respiration.

### 2.2. In Vitro Small Airway Models Using Organ-on-a-Chip

Inspired by the alveolar chip, a small airway-on-a-chip with a similar structure was designed to study the physiological state of the human small airway and related diseases. Benam et al. reported a classic small airway-on-a-chip, which is similar to the above model. It consists of dual-chamber microchannels in which the primary airway epithelial cells and microvascular endothelial cells are cultivated on both sides. Interestingly, the Benam’s team successfully induced the differentiation of airway epithelial cells by removing apical medium after five days of primary airway epithelial cell culture and adding retinoic acid (3 μg mL^−1^) into the inferior microchannel. In this model, ciliated epithelial cells, mucinous goblet cells, club cells, and basal cells were co-cultured successfully, and their proportions were similar to those of normal human lungs. In addition, the team also perfused IL-13 in the inferior channel to rebuild a microenvironment of asthma, which was rich in helper T cells (Th2), goblet cells, and pro-inflammatory factors [26,27]. This model has a simple structure and a high degree of resemblance in the differentiation of airway epithelial cells in vivo, so it has a milestone significance in the field of small airway-on-a-chip.

Subsequently, on the basis of this model, Benam et al. designed a smoke-generating device and integrated it on the small airway-on-a-chip. Smoke was introduced into the upper microchannel of the chip to observe the response of epithelial cells, such as ciliary micropathology and COPD-specific molecular characteristics [25]. Recently, Janna et al. introduced a virus into the model and described a rhinovirus-induced asthma exacerbation model. Specifically, IL-13 was used to stimulate fully differentiated mucociliary airway epithelial cells to induce the Th2 asthma phenotype and infection with rhinovirus 16 (HRV16). The viral infection of the asthmatic airway epithelium and migration of neutrophils were reproduced, and immunomodulatory therapy could be evaluated [28].

In addition, the three-chamber microchannel structure of small airway-on-a-chip is also widely used. Unlike the above-mentioned two-chamber channel model, an additional compartment between the airway epithelial channel and the microvascular endothelial channel is inserted, containing fibroblasts [29] or extracellular matrix components [30]. The platform is expected to study the cross-communication between three different cells and their respective effects on each growth and differentiation. Moreover, small airway-on-a-chip that includes fibroblasts may be meaningful for studying diseases such as idiopathic pulmonary fibrosis. 

### 2.3. Organ-on-a-Chip Simulates Cellular Interaction in the Alveoli

There is also a wide range of fibrous matrix components and interstitial cells in the alveolar compartment, such as alveolar macrophages and fibroblasts [31], which endow most mechanical properties of the tissue. They also modulate alveolar epithelial cells by releasing certain signaling molecules in response to infection, epithelial barrier damage, and pathogen clearance [32]. Microfluidic technology allows spatial control of fluids in micron-sized channels. We can co-cultivate a variety of cells, generate and control gradients of signal molecules, and realize dynamic perfusion culture.

In 2005, Rothen Rutishauser et al. designed a co-culture model to simulate the cellular part of the alveolar–capillary barrier, which includes macrophages, alveolar epithelial cells, and dendritic cells. Alveolar epithelial cells (A549 cells) were cultured on membrane filter inserts with a pore size of 3 μm. Human macrophages and dendritic cells were co-cultured on the apical and basal sides, respectively [33]. Although this model uses confocal laser scanning and conventional transmission electron microscopy to study the interaction of alveolar epithelial cells with macrophages and dendritic cells during the ingestion of polystyrene particles (1 μm in diameter), there are no vascular endothelial cells in the model, which makes the barrier incomplete. Next, Luyts et al. introduced vascular endothelial cells into the model and established a co-culture model using human bronchial epithelial cells (16HBE14o-), monocytes (THP-1), and human lung microvascular endothelial cells (HLMVEC). In this model, functional tight junctions are expressed, and the transepithelial resistance (TEER) value is sufficiently high. Interestingly, with the addition of mononuclear macrophages, TEER was reduced by 73% [34]. In addition, Humayun et al. have developed a small airway-on-a-chip based on thermoplastics for cell–cell interactions, which are between airway smooth muscle cells (SMCs), airway epithelial cells (ECS), and the extracellular matrix. The model consists of three-chamber microchannels, including a bottom microchannel for SMCs, a thin hydrogel layer in the middle, and an upper microchannel for the air–liquid interface (ALI), in which co-culture was realized for more than 31 days [35]. Punde et al. reported a microsystem based on the model mentioned above to monitor the role of eosinophil cationic protein (ECP) in lung inflammation. In the inflammatory state, ECP induces airway epithelial cells to express CXCL-12, which stimulates the migration of fibroblasts to the epithelium. The microsystem can mimic the cross-cell migration under the physiological flow in vivo [36]. However, the cells are all immortalized cell lines in the above models, but the primary cells are closer to the state in vivo. Sellgren et al. designed a microfluidic model in which the cells used in the model are all primary cells of the human airway. Since then, airway epithelial cells have shown better mucociliary differentiation and barrier function [29]. 

In addition, lung-on-a-chip also provides suitable conditions for observing cellular differentiation. The lung-on-a-chip can monitor the changes in the number, composition, appearance, and structure of cells in the alveolar or airway epithelium in vitro in real-time. Many studies have shown that when the airway epithelial is in culture alone, the TEER value increased significantly and peaked during the first week, while in air–liquid interface (ALI) culture, TEER decreased steadily. This phenomenon may be due to the formation of a moderately leaky epithelial layer, which has good basal apical transport and secretion functions [37,38]. Cozens et al. showed that the initial stage of ALI culture was dominated by cell proliferation, and the cells began to differentiate after the number of cells was sufficient. These processes are coordinated and interleaved and last about a week [39]. Benam et al. reported a small airway-on-a-chip that induced differentiation of airway epithelial cells by removing apical culture medium and adding tretinoin. The final chip contains well-differentiated mucociliary bronchiolar epithelium and pulmonary microvascular endothelium. In addition, the team added interleukin-13 (IL-13) to the epithelium in order to induce the proliferation of goblet cells and the excessive secretion of cytokines. They successfully reconstructed the airway response of asthma patients and made it easier to analyze lung pathophysiology at the organ level [26]. 

### 2.4. Organ-on-a-Chip Simulates Mechanical Strain in Respiratory Movements

After Huh’s team, Stucki et al. created a new lung-on-a-chip platform [40] in which the simulation of a mechanical strain of respiratory movement was also realized. Inspired by the diaphragm movement during respiration in the body, the platform proposed a three-channel model representing the lung parenchymal environment, which can simulate the three-dimensional strain caused by respiratory movement (Figure 4b). In this model, the respiratory movement is generated by applying a very small pressure to the medium at the basal side of the porous membrane. This pressure can drive the ultrathin porous membrane, causing the alveolar epithelial cells and endothelial cells to undergo respiratory movement. The design of the model is not only to reproduce the environment in the lung parenchyma but also to make it stronger and easier to handle. In addition, the model puts forward the concept of reversible bonding, enabling it to precisely control the concentration of cells cultured on the membrane. In this model, it is found that the circulatory stretching caused by respiration can significantly affect the permeability of the epithelial cell layer and regulate the secretion of surfactants in alveolar type II epithelial cells, which has also been confirmed in other related studies [41,42]. Furthermore, the team used the same ultrathin porous membrane to culture primary cells and designed a similar middle-throughput alveolar barrier model [43]. Subsequently, Zamprogno et al. improved the ultrathin porous membrane made of collagen and elastin. It uses a thin gold mesh with a pore size of 260 μm as a scaffold, which can replicate 40 stretchable films to stimulate a three-dimensional strain in the body, which realizes the high-throughput simulation in the lung-on-a-chip [44]. Additionally, subsequent research has used other materials to improve the porous membrane to close the physiological thickness and pore structure and reduce its absorption of small molecules. Mondrinos et al. introduced a new type of porous membrane for cell culture, which is made of a natural extracellular matrix and is easy to integrate into a microfluidic device [45]. Yang et al. developed a new lung-on-a-chip using polylactic–glycolic acid (PLGA) electrospun nanofiber membrane as the matrix and cell scaffold. In this model, human non-small cell lung cancer cells (A549), human fetal lung fibroblasts (HFL1), and human umbilical vein endothelial cells (HUVEC) were co-cultured, and the immune infiltration of non-small cell lung cancer was observed [46]. In addition, Mermoud et al. reported a new micro-impedance tomography (MITO) system, which can be integrated with the lung chip and can use coplanar impedance electrodes to monitor the electrochemistry changes and mechanical changes that occurs in the alveolar barrier at a distance of 1 mm. The study showed that when the porous membrane is deflected to mimic respiratory movement, the impedance changes and is related to the mechanical strain in the alveolar barrier [47]. Huang et al. designed an alveolar lung-on-a-chip platform using a hydrogel made of gelatin methacryloyl with an inverse opal structure. This material has well-defined and interconnected pores that are highly similar to human alveolar sacs. By culturing primary human alveolar epithelial cells on the chip, the functional alveolar epithelium can be easily formed, and breathing movement can be realized [48].

### 2.5. Organ-on-a-Chip Simulates Alveoli and Small Airway Anatomy

Most of the lungs-on-a-chip introduced above use traditional manufacturing technology to form a fixed-shaped PDMS to create a channel with a rectangular cross section. However, based on the characteristics of the lung anatomy in the human body, a circular cross-section channel may more accurately simulate the fluid dynamics in the alveoli and small airways. Anatomically, the lung is composed of a large bifurcated tree-like structure. The bronchus of the lung branched for 23 times into bronchioles of different grades [49]. Their ends expand into sacs and small vesicles called alveoli. Alveoli are hemispherical vesicles composed of a single layer of epithelial cells. They first appear around the 17th level of the tree-like structure. The total number of alveoli in an average adult is usually more than 30,000 and the volume of alveoli varies from 50 to 450 mm^3^, with an average of 187 mm^3^ [50]. The shape of alveoli is similar to spheres, ellipsoids, and cylinders [51], and the gas exchange zone composed of many alveoli is similar to a honeycomb structure composed of hollow polyhedral cavities [52]. Due to the unique properties of PDMS, complex geometric shapes can be generated from soft materials, which can more faithfully replicate the morphology of alveoli and small airways in the body and restore the respiratory particle dynamics in the alveoli under physiological conditions. 

In the simulation of alveolar morphology, Fishler et al. constructed a lung-on-a-chip with the real size of the body on which the locus of the particles can be directly observed and detailed deposition locations can be drawn (Figure 4c). The chip contains five generations of bifurcated bronchi, the channel width is reduced from 345 to 120 μm, and it can be periodically changed with respiratory movement. This model reconstructs the locus of smoke particles near the alveolar cavity, which is of great significance for studying the dispersion and deposition of inhaled particles and drugs in the alveoli [53]. Unlike traditional microfluidic devices that require stacking multiple PDMS molds, they proposed a simple method for manufacturing the top cavity by embedding the syringe barrel partly into the PDMS mold. This novel design can provide physiological respiratory movement, which generates airflow similar to that under physiological conditions [54,55]. In addition, Katan et al. designed a chip to study the flow topology in the fetal lung at the embryonic stage, reproducing the true proportions of the fetal airway in three different pregnancy stages and revealing the unique flow patterns at different stages of fetal life [56].

In addition, the current inhalation model in vitro cannot simulate the intricate bronchi network for many toxic gases and harmful dusts. It is still challenging to determine reliable deposition results quantitatively for various gravity directions (such as tip and basal lung lobes) and different anatomical constraints (such as circular cavities and alveolar cavities), and it is more difficult to satisfy the mechanism research and specific treatment of acute and chronic injury. The lung-on-a-chip is an attractive way to improve the inhalation physiological toxicology model in vitro. 

In the simulation of small airway morphology, Nesmith et al. established a deformable bronchial smooth muscle tissue elastic film, which vividly simulates bronchoconstriction and expansion (Figure 4d). The team found that exposure to IL-13 can cause high-strength contraction and induce film bending, while exposure to muscarinic antagonists and β-receptor agonists can reverse this contraction, which is similar to clinical observations and animal tissue studies. This chip provides an important tool that can evaluate airway smooth muscle at the level of protein expression, tissue structure, and function in vitro to find new therapeutic targets [57].

In general, the lung-on-a-chip based on microfluidic technology can simulate the microenvironment of the human body to carry out complex metabolism and regulation. It can provide a new technology platform for further study in many aspects including the alveolar–capillary barrier, cell composition, and differentiation conditions and intercellular communication, which have been widely used to explore the mechanism of various physiological processes in the lungs. 

## 3. Pathological Model of Respiratory System Based on Organ-on-a-Chip

In addition to being in charge of respiratory function, the lungs also have defense, immunity, endocrine, and metabolic functions. The lungs are connected to the outside atmosphere, which can be called the first barrier of the human body, and blood flows through the lungs for gas exchange, which makes the lungs suffer the invasion of microorganisms in the external environment [58]. Once the immune balance of the lungs is broken, a series of pathological reactions will occur. When the amount of virulence of infectious pathogens entering the lung exceeds the immune tolerance, it will trigger an “inflammatory factor storm” in the lungs, leading to infectious pneumonia. When the lung is attacked by external allergens, it will lead to bronchial asthma because of protective immune and allergic reactions. When the respiratory system is stimulated by air pollutants or smoke for a long time, it will cause bronchi remodeling and lead to obstructive lung diseases such as emphysema [59]. In these diseases, environmental factors cooperate with immune cells in the airway to produce different phenotypes, which makes it very complicated to study in vitro.

In traditional cell experiments, the co-culture of multiple cells is not easy to achieve, and the alveolar epithelial cells are in a state of dynamic mechanical stretching for a long time under the influence of respiratory movement. Two-dimensional cell culture in vitro cannot simulate the physiological state, and this mechanical strain change will greatly affect the transportation of hydrophilic molecules.

In addition, the construction of animal disease models is also commonly used in vitro to study pathogenesis. In fact, animals do not naturally have diseases, and even if they can obtain a certain disease performance through intervention in the external environment, they cannot summarize all aspects of human pathogenesis [60]. This may be due to the differences in airway physiology, anatomy, and immunology between animals and humans. For example, for the simulation of bronchial asthma, because the abundance of goblet cells in mice is much lower than that in humans, it cannot simulate the pathological state of goblet cell proliferation during an acute exacerbation of asthma [61].

All these make it very difficult to study the pathogenesis of complex diseases in vitro. The emergence of the organ chip provides a new way to solve the above problems. It can integrate the organ-specific 3D microenvironment, tissue–tissue interface, dynamic mechanical strain, vascular perfusion, and potential function of immune cells. In the following, we will discuss several diseases that may benefit from lung-on-a-chip. 

### 3.1. Acute Lung Injury and Acute Respiratory Distress Syndrome

Acute lung injury (ALI) is the damage of alveolar epithelial cells and capillary endothelial cells caused by various direct and indirect factors [62], leading to diffuse pulmonary interstitial and alveolar edema, extensive infiltration of neutrophils, and even acute hypoxic respiratory insufficiency [63,64]. The clinical manifestations are progressive hypoxemia and respiratory distress. Acute respiratory distress syndrome is accompanied by surfactant dysfunction, which makes it challenging to reduce surface tension. In addition, the mucous film covering the epithelium of the small airways is more prone to gas–liquid instability. This condition usually results in the formation of a liquid plug in the lumen of the airway, which blocks the small airway and gas exchange in the alveoli. The expansion of the lungs during inhalation causes the liquid plug to spread and rupture, thereby reopening the blocked airway [65]. Because the special membrane structure of alveoli makes it highly sensitive to mechanical strain, abnormal strain changes may cause or aggravate lung injury [62,66]. As an important part of the lung, the degree of alveolar epithelial injury will significantly impact the process of lung injury.

**Figure 5:**
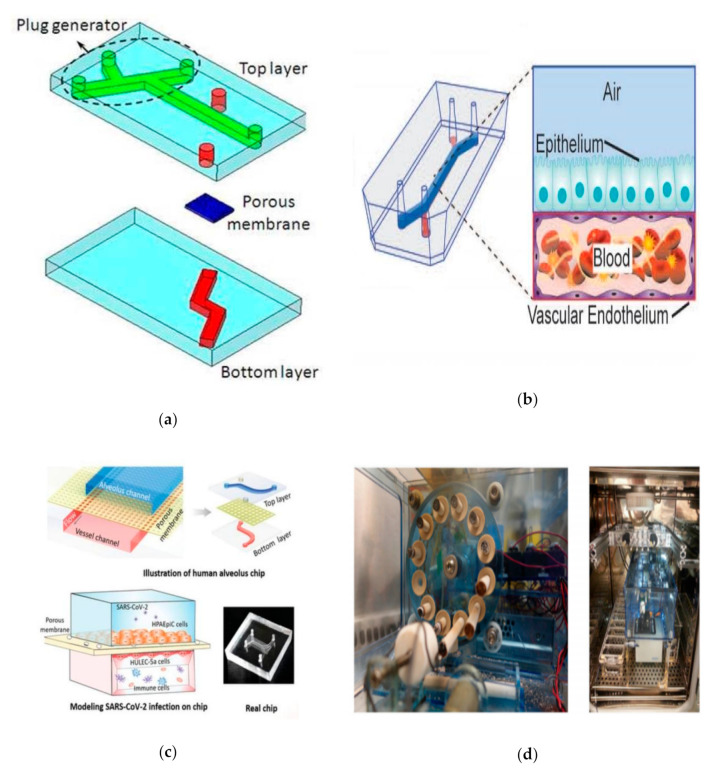

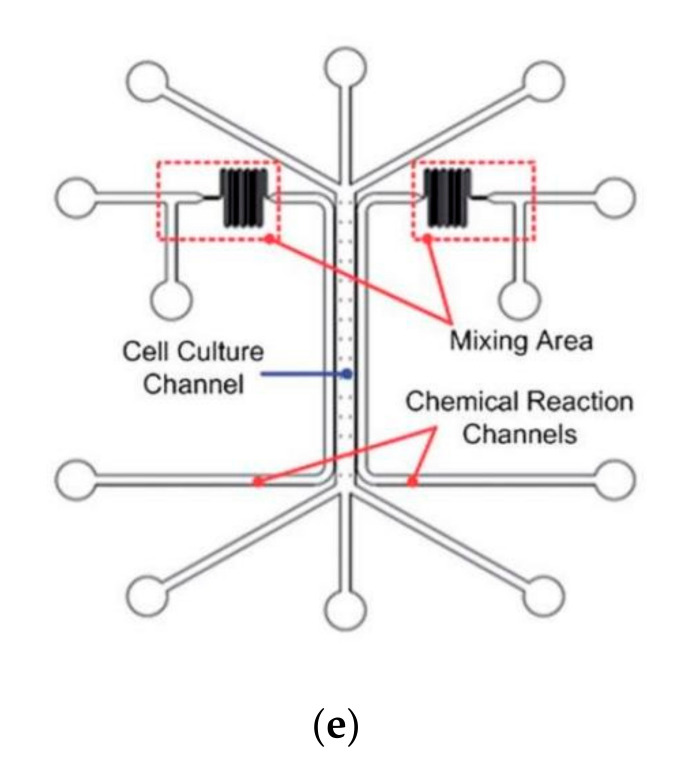
Several pathological models of the respiratory system based on lung-on-a-chip: (**a**) the microfluidic chip for studying formation of liquid plugs produced by Tavana et al.; (**b**) the lung-on-a-chip for quantitative analysis of inflammation-mediated thrombosis at the organ level produced by Jain et al.; (**c**) the lung-on-a-chip in which virus infection of alveolar epithelial cells was observed produced by Qin et al.; (**d**) the lung-on-a-chip that simulates smoke-induced COPD produced by Benam et al.; (**e**) the microfluidic chip in which oxygen concentration gradient was realized produced by Chen et al.. Reproduced with permission from [24,25,71,84,97].

Bilek et al. designed a fluid-filled channel structure lined with epithelial cells. A semi-infinite bubble is formed by injecting gas into the channel to empty the liquid, which can simulate the sudden opening of the obstructed airway caused by acute respiratory distress syndrome. In this model, epithelial cell damage caused by changes of mechanical strain can be observed. Moreover, the damage degree of epithelial cells is directly related to the pressure gradient near the tip of the bubble [67], and has nothing to do with the time of pressure [68]. In addition, Yalcin et al. studied the effects of airway reopening speed and airway diameter on epithelial cell damage. In the process of reopening the airway by mechanical ventilation in acute respiratory distress syndrome, the distal area of the lung was more likely to be injured, and rapid inflation may have a cytoprotective effect [69]. Based on this, Huh et al. studied the mechanical damage of primary human small airway epithelial cells caused by the movement of a finite-length liquid plug in three-dimensional microfluidic system. The fluid embolism was generated by an embolization generator integrated into the upper cavity channel, and it would gradually shorten when moving down the microchannel and eventually rupture in the downstream area. When the liquid plug becomes very thin and ruptured, there was a higher risk of mechanical damage to the epithelial cells [70]. Furthermore, Tavana et al. studied the factors affecting the formation of liquid plugs. When surfactants were added to the lung-on-a-chip, there were significant differences in the shape and diffusion of the liquid plugs (Figure 5a) [71].

### 3.2. Pulmonary Embolism

The phenomenon that the abnormal substances insoluble in the blood appear in the circulating blood and travel to a distant place with the blood flow to block the vascular cavity is called vascular embolism. Due to the anatomical characteristics of the pulmonary artery and local blood circulation, more than 90% of deep vein thrombosis and right heart mural thrombosis will stay in the pulmonary artery [72]. If the embolus is too large or pulmonary congestion occurs before the embolism, it may cause serious consequences such as pulmonary hemorrhagic infarction or sudden death. Therefore, thrombus formed by platelet activation and inflammation is the main cause of death in many patients with lung diseases [73,74]. However, in traditional animal models, it is difficult to study the platelet–endothelial interactions and epithelial–endothelial interactions that can promote the development of thrombosis, while organ-on-a-chip can make up for this deficiency.

Jain et al. first proposed a microfluidic device that simulates the vascular network of stenotic arterioles, evaluating blood coagulation in small sample sizes under pathological conditions. The coagulation and platelet function can be accurately measured in vitro by analyzing the coagulation time based on the phenomenological mathematical model of thrombosis [75]. Based on this, the team designed an alveolar chip, arranging vascular endothelial cells on the wall of the microchannel to create a vascular cavity. It can be used to quantitatively analyze inflammation-induced thrombosis at the organ level by perfusion of human blood instead of culture medium (Figure 5b). Stimulating vascular endothelial cells with TNF-α before initiating blood flow caused rapid platelet recruitment, consistent with the formation of microvascular thrombosis in vivo [24]. This chip summarizes platelet–endothelial dynamics and reveals that lipopolysaccharide (LPS) endotoxin indirectly stimulates intravascular thrombosis by activating alveolar epithelium [24]. In addition, as an emerging material, the hydrogel is widely used in studying the physiology of blood vessel-related cells and constructing relatively large 3D tissues, because it can better mimic the vascular tissue-like structure in terms of morphology. Kinoshita et al. built a vascular tissue model with multi-layered, branched, and thick-walled structure by forming a hydrogel in situ in the fluid microchannel and introduced vascular endothelial cells (ECs) and smooth muscle cells (SMCs) into in vitro culture for seven days to form a multi-layered vascular tissue [76]. Peak et al. used hydrogel to co-culture primary human vascular endothelial cells and fibroblasts to achieve the construction of a 3D vascular network. The network adopted an open-chamber design that can introduce lung adenocarcinoma cells to form a vascularized model. The 3D model of lung adenocarcinoma can be used as a high-content drug screening platform to simulate intravascular delivery, tumor-killing effects, and clinical chemotherapeutics’ vascular toxicity [77]. In addition, Nie et al. innovated the hydrogel material, using a combination of gelatin and gelatin methacrylate (GelMA) to make it have the best biocompatibility. The new material and microfluid technology provide a promising model for further research on tissue engineering and drug development of blood vessel formation and vascular inflammation [78].

### 3.3. Lung Inflammation and Infectious Diseases

Lung inflammation is a common lung disease usually caused by infection with bacteria, viruses, mycoplasma, fungi, and other pathogens (such as parasites) [79]. When the amount of virulence of infectious pathogens entering the lungs exceeds the immune tolerance of the lungs, the immune homeostasis in the lungs will be unbalanced, triggering excessive inflammation, such as the “cytokine storm” after viral or bacterial infections, which may cause extensive lung tissue damage [80]. The interaction mediates the pathogenesis of respiratory diseases between the infectious pathogens and the host immune system (including lung resident cells and circulating immune cells) to a large extent. Because the lung-on-a-chip can allow immune cells to dynamically pass through the pulmonary capillaries, it provides an unprecedented possibility for studying the interaction between lung endothelial cells and epithelial cells.

Huh et al. were pioneers in creating a pulmonary inflammation model, introducing pro-inflammatory cytokines (tumor necrosis factor-α) and bacteria (*E. coli*) into the alveolar cavity on the upper layer of the alveolar–capillary interface. The team increased the expression of intercellular adhesion molecule-1 (ICAM-1) on the other side of porous membrane, which promoted the formation of capillaries and simulated the adhesion of neutrophils in the microchannels on the surface of vascular endothelial cells to achieve the recruitment. In addition, neutrophils were observed in this model to migrate and penetrate vascular endothelial cells into the alveolar microcavities and to phagocytose *E. coli* [22]. Zhang et al. described a microfluidic platform at the alveolar–capillary interface, which can comprehensively evaluate lung inflammation and damage caused by environmental pollutants. This platform confirmed the epithelial and endothelial synergy in the pulmonary inflammatory state [81]. Next, Punde et al. added fibroblasts to the model to monitor the role of eosinophil cationic protein (ECP) in lung inflammation. They found that ECP can induce the expression of CXCL-12 in airway epithelial cells and then stimulated the migration of fibroblasts to epithelial cells, revealing the role of the CXCL12–CXCR4 axis in mediating ECP-induced fibroblast extravasation in pneumonia [36]. Therefore, it is clear that the organ-on-a-chip in vitro is of great significance for exploring the interaction between pulmonary infectious diseases and resident immune cells. In addition, this unique feature can also be used to analyze the underlying disease mechanism of special bacterial infections, such as tuberculosis infection. Thacker et al. used primary mouse cells to explore the direct role of lung surfactants in early tuberculosis infection. Surfactant deficiency led to the rapid and uncontrolled growth of bacteria in alveolar cavicity, which was essential for finding a treatment for tuberculosis [82]. COVID-19 caused by SARS-CoV-2 infection caused a global health crisis in 2020. Qin’s team used lung-on-a-chip to characterize the response of alveolar epithelium and adjacent microvascular endothelium to viral infections in the co-culture system. Studies have shown that virus infection first replicates in alveolar epithelial cells, then undergoes dramatic mitochondrial remodeling, and then affects endothelial cells through unrecognizable crosstalk, resulting in alveolar–capillary damage during infection [83]. Furthermore, the team introduced circulating immune cells into the chip model and observed the recruitment of immune cells, endothelial detachment, and increased release of inflammatory cytokines caused by infection, suggesting that immune cells participate in alveolar barrier damage and exacerbate inflammation (Figure 5c) [84].

### 3.4. Obstructive Lung Disease

COPD is the most prevalent chronic respiratory disease in the world. It is related to inflammatory response by harmful gases and particles. The global incidence rate of COPD is as high as 9–10% for people over 40 years old, and it can further develop into pulmonary heart disease and respiratory failure [85]. The chronic inflammation is closely related to viral and bacterial infections in the respiratory tract. Although inhaled drugs can be used to control symptoms, there is still no effective method to reduce disease progression, because the existing models cannot accurately characterize the impact of environmental factors and circulating immune cells on obstructive lung disease. Microfluidic technology can overcome these two shortcomings. Lung-on-a-chip can help dynamically analyze environmental factors and provide a platform for dynamic interaction between lung tissue and circulating immune cells. 

Benam et al. proposed a small airway chip that contains differentiated mucociliary bronchiolar epithelium. The chip had almost the same cell proportions as those in normal human lungs, including ciliated epithelial cells, goblet cells, club cells, and basal cells. After stimulation by interleukin-13 (IL-13), goblet cell proliferation, high secretion of selective cytokines, and increased recruitment of neutrophils could be observed in the chip, successfully realizing the simulation of the pathological state of asthma [26]. On the basis of this model, the team designed a device that can be connected to the chip. The device could “inhale” different amounts of cigarette smoke into the chip. It could also be programmed automatically to summarize human smoking behavior in vitro and study the pathophysiology of smoke-induced COPD (Figure 5d) [25]. In addition, the use of this model has also achieved the simulation of acute exacerbations of asthma and COPD. When airway epithelial cells were stimulated by bacterial, lipopolysaccharide endotoxin (LPS), or Poly I:C (viral mimics), M-CSF and IL-8 secreted more in the chip compared to in the healthy lung. M-CSF promotes the differentiation and survival of macrophages, while IL-8 is an inducer of neutrophils, both of which are the main immune cell types in patients with asthma and COPD [86,87]. Nesmith et al. established a layered bronchial smooth muscle tissue on an elastic film through microfluid technology to simulate the bronchoconstriction and expansion of asthma patients in vitro. The contraction of the muscle layer caused by cholinergic agonists and the bending of the chip film could be observed on the chip, and cholinergic antagonists and β-receptor agonists, which are clinically used to relax narrow airways, have been used to reverse this process [57]. These powerful methods for simulating chronic inflammation of the human lung in vitro are of great significance for detecting the synergistic effect of lung epithelial cells and vascular endothelial cells on the secretion of cytokines, identifying new biomarkers of disease progression and detecting the response of disease anti-inflammatory compounds.

### 3.5. Lung Cancer

Lung cancer is one of the malignant tumors with the fastest increase in morbidity and mortality and the greatest threat to health [88]. In the past 50 years, many countries have reported that the incidence and mortality of lung cancer have increased significantly, and the mortality of lung cancer in men accounts for the first place in all malignant tumors [89]. Although the existing treatment methods such as surgery, radiotherapy, and chemotherapy can improve the survival rate, the drug resistance of tumor cells and the ultimate fatal metastasis are still widespread. The organ-specific microenvironment plays an important role in determining tumor biology and drug response. The colonization and neovascularization of circulating tumor cells in other organs are closely related to tumor metastasis [90], but it is difficult to achieve those in traditional 2D in vitro culture and other cell experiments. Therefore, there are few in vitro studies on tumor metastasis and drug response. In addition, emerging immunotherapies including antibody-based immune checkpoint receptor inhibitor therapy are a promising treatment strategy that can cause anti-tumor immune responses [91]. However, some immune checkpoints do not exist in animal models, which cannot completely replace human experiments. Therefore, immune checkpoint receptor inhibitors are difficult to translate into preclinical efficacy [92]. The lung-on-a-chip provides a new strategy for the above problems. It can simulate the unique 3D microenvironment of internal organs and the potential interaction between tumor cells and immune cells. In addition, it can also continuously monitor the evolution of tumors and the interaction with other organs.

Hassell et al. created an in situ model of non-small-cell lung cancer (NSCLC) to summarize the growth and invasion in the organ-specific microenvironment. In addition, breathing movement was also introduced into the model to observe the tumor dormancy under mechanical strain and the response to tyrosine kinase inhibitor (TKI) treatment [23]. Jung et al. proposed a one-stop microfluidic device that can directly perform 3D lung cancer organoid culture on the microphysiological system (MPS) and achieve drug sensitivity testing at the organ level. It was observed that the standard treatments for lung cancer, cisplatin and etoposide, increased the induction of apoptosis in a concentration-dependent manner. However, the organoids still contained drug-resistant tumor cells in the core, which could be used to guide preclinical levels treatment [93]. Ruzycka et al. summarized a new type of personalized nanomedicine printable material to study the microfluidic system of lung cancer metastasis, tumor cell growth, endothelial cell migration, and finally, angiogenesis. Furthermore, they discussed intercellular crosstalk between lung cancer cells and surrounding environmental cells and the connection with various molecular signals from the external cell matrix [94]. Liu et al. developed a multi-organ microfluidic chip as a new platform for studying brain metastasis of lung NSCLC. The chip was composed of two bionic organs, the upstream “lung” and the downstream “brain”. It had a functional “blood–brain barrier (BBB)” structure, so that it could be observed in real-time that the primary tumor breaks through the BBB and finally reached the brain parenchyma to achieve brain metastasis. Additionally, the chip could be used to verify the metastasis ability of different lung cancer cells [95]. In addition, Xu et al. conducted a proteomic study on the chip. They found that the expression profile of metastatic cancer has undergone tremendous changes compared with primary lung cancer cells. Expression of GSH metabolism-related enzymes, glutathione peroxidase, and aldehydes were increased, which provided a potential target for metastatic brain cancer to solve the problem of drug resistance [96]. In addition, research on the biological behavior and drug treatment of lung cancer in hypoxic microenvironment is also a research hotspot of microfluidic technology. Chen et al. designed a microfluidic device in which the oxygen concentration gradient was realized through chemical reactions. Then, carcinomic human alveolar basal epithelial cells (A549) were cultured with an anti-cancer drug (tirapazamine) under different oxygen gradients, and they demonstrated the hyperoxia-induced cell death and hypoxia-induced cytotoxicity of tirapazamine (Figure 5e) [97]. Coincidentally, Wang et al. and Chang et al. also generated an oxygen concentration gradient in a single microfluidic device and demonstrated the anti-tumor function of tirapazamine, respectively [98,99]. Meanwhile, Chang et al. also proved that the oxygen gradient plays an essential role in guiding cell migration [99]. In recent years, some studies have begun to use microfluidic devices to explore specific molecular functions in hypoxic environments. Jin et al. used a microfluidic chip to explore the effect of Netrin-1 on the epithelial-to-mesenchymal transition of A549 cells in the hypoxic microenvironment [100]. With the maturity of hypoxic condition control technology, microfluidic devices will be more widely used.

## 4. Application and Prospect of Respiratory Organ Chips in Reality

**Figure 6:**
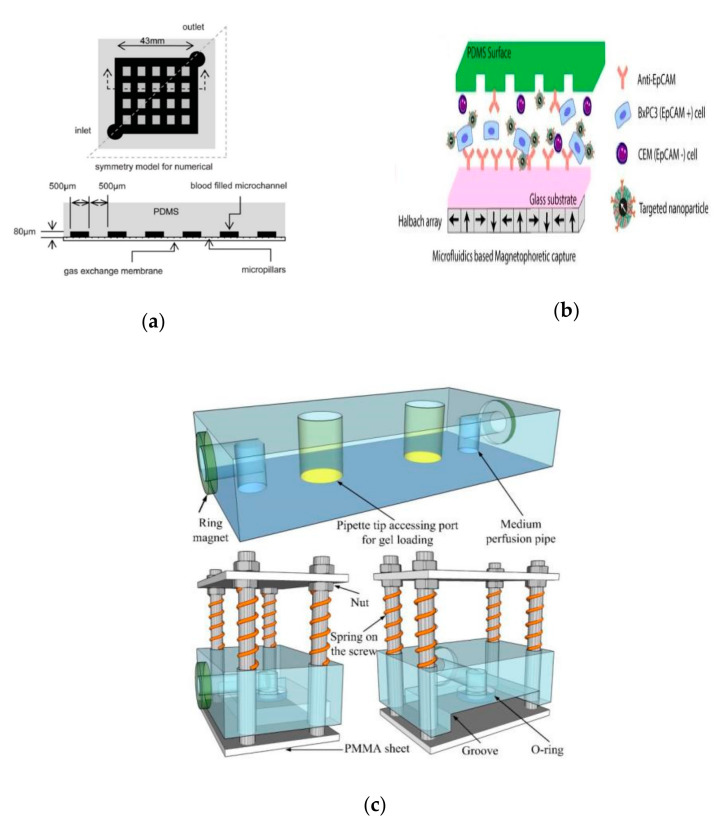
Several application examples of respiratory system based on lung-on-a-chip: (**a**) the microfluidic device for auxiliary gas exchange produced by Sun et al.; (**b**) the microfluidic capture device integrated with magnetic nanoparticles; (**c**) the reusable standardized universal interface module produced by Sun et al. Reproduced with permission from [102,104,111].

The distinctive advantages of organ-on-a-chip provide a better technical platform for scientific research on human diseases. The size of its microchannel can be similar to the space for cell growth in vivo. It can form a complex multi-dimensional structure under sophisticated design to simulate the real anatomical structure of a tissue or organ. For example, the circular alveolar cavity designed on the chip more realistically simulates the physiological conditions in vivo. On the other hand, microfluidic technology is also under systematic study to provide viable platforms for auxiliary gas exchange in vitro. Compared with the traditional hollow fiber oxygenator, the microfluidic device can be closer to the physiological gas exchange process and has a natural advantage in the surface-to-volume ratio of gas exchange. Gas transmission efficiency and flow resistance are important parts of the related research. Similar to what people imagined, oxygen transfer capability is mainly affected by membrane thickness, channel depth, network design, and blood flow rate [101]. Through comparing four different membranes, including thin film PDMS, porous PDMS, and two different pore size porous polycarbonate membranes, Wu et al. found that the microfluidic oxygenator with porous PDMS membrane has the highest gas exchange rate of 1.46 μL min^−1^ cm^2^ for oxygen and 5.27 μL min^−1^ cm^2^ for carbon dioxide and performs better than a commercial hollow fiber-based oxygenator by 367% and 233%, respectively. They also proved that the pressure drop increases with the flow rate, and the pressure drop in the oxygenators with PDMS membranes is generally lower than that with porous polycarbonate membranes at the same flow rate (Figure 6a) [102]. After studying the influencing factors of oxygenators, Kniazeva et al. suggested that high levels of oxygen transfer, low blood prime volumes, and improved blood flow are necessary for future microfluidic oxygenators [101]. In addition, organ-on-a-chip can achieve long-term co-cultivation of multiple cells, providing an ideal platform for studying the interaction between cells. It also has outstanding advantages in the simulation of tissue contact interfaces, such as the vascular endothelial cell–peripheral cell interface simulation. The organ-on-a-chip based on microfluid technology can produce precisely controllable fluid shear force, periodically changing mechanical force, and perfusate with varying solute concentration gradients. In the present SARS-CoV-2 pandemic, microfluidic technology also provides the possibility for rapid and accurate virus detection. A reciprocating–flowing on-a-chip was designed for detection of SARS-CoV-2 antibodies. The antibodies fully contact the antigen through repeated flow in the chip to improve the accuracy of the detection [103]. Additionally, it is reported that the microfluidic capture device integrated with magnetic nanoparticles can capture specific tumor cells and even viruses in the cell mixture (Figure 6b) [104]. Furthermore, Si et al. designed a microfluidic bronchial-airway-on-a-chip lined by highly differentiated human bronchial-airway epithelium and pulmonary endothelium, which can quickly identify candidate antiviral therapeutics and preventives [105]. To sum up, the chip can be used to analyze the specific pathophysiological changes of tissues and organs meticulously, and therefore it is widely used in scientific research on human diseases.

However, there are few cases where organ chips are actually used in real life or clinical treatment. This is because there are some non-negligible defects that prevent the commercialization of organs-on-chips. Although organ-on-a-chip can better simulate the microenvironment, as described above, large-scale and low-cost production is more difficult due to these characteristics. In addition, there are still some controversies in the use of materials and manufacturing methods. Traditional PDMS materials have some limitations in scientific research and actual applications. It is more important that PDMS can absorb hydrophobic small molecules, which may affect the detection of certain drugs on the platform [106]. In addition, due to the high cost of materials and complex production process, mass production is difficult. Recently, thermoplastic materials, represented by polymethyl methacrylate (PMMA), have emerged due to their simple manufacturing and low cost. As an alternative to PDMS, PMMA is a low-cost material that can be mass-produced by laser cutting machines. Busek et al. thermally combined thermoplastic elastomer (TPE) films with laser-cut PMMA sheets to build a multilayer microfluidic device with integrated pneumatic micropumps [107]. In addition, the latest research has proposed a new type of thermoplastic elastic material, which is composed of flexdymtm polymer and can withstand pressures of 500 mbar and above. It can be quickly assembled into a microfluidic device with a commercial porous polycarbonate membrane [108].

Furthermore, when adding specific functions to the chip system, such as respiratory mechanical movement, flow control of complex multi-dimensional structures, etc., it is undoubtedly necessary to conduct special training for the user [109]. In this respect, more mature in vitro co-culture models (such as Transwell, etc.) have more tremendous advantages. The Transwell co-cultivation model only contains well plates, Transwell inserts, and porous membranes [110]. The realization of cell co-cultivation in this system is almost the same as the traditional cell culture steps. Although Transwell cannot achieve a more simulated in-vivo microenvironment, it is more widely used in cell co-cultivation due to its design simplicity, stronger repeatability, and operability. Moreover, it is easier to commercialize mass production. Therefore, in the future applications of respiratory organ chips, efforts should be made to achieve a compromise between simulating the complex in-vivo microenvironment and user-friendliness in order to solve the most important biological problems. Researchers have also made some attempts to solve the above shortcomings. Current research focuses on modularizing single-organ chips to simplify the integration of multi-organ models, which provides users with an intuitive and flexible way to configure different multi-organ systems. Sun et al. proposed a reusable standardized universal interface module, which is implemented in a plug-and-play manner and avoids fluid leakage and can be well used for the integration of multi-organ systems. Its flexible design makes it simple to integrate multiple modular single-organ chips into a high-throughput platform, which can greatly reduce the cost of commercialization (Figure 6c) [111]. In addition, some interface modules with specific functions have also been designed. Louis et al. designed a tissue and fluid control module with self-aligning magnetic interconnection to solve fluid leakage and alignment between individual monolithic modules [112]. Satoh et al. built a new type of multi-flux multi-organ chip system with the help of a medium circulation platform driven by air pressure [113]. Nie et al. designed a module driven by capillary force and a simple and efficient manufacturing process of microfluidic channels through 3D printing [114]. In short, through the above modular interface, users can combine different organ-on-a-chip modules to achieve a variety of different functions. The single module can also be taken out for testing and analysis. Single-organ chip modularization greatly accelerates its commercialization process.

In the future, if commercial organ chips can be applied to the clinical treatment process, it is expected to completely change the treatment and rehabilitation of patients with lung diseases. In fact, due to the complexity and controllability of the structure of organ-on-a-chip, specific individual cells, tissue samples, and culture parameters can be controlled and integrated, which is very suitable for directly simulating the physical and chemical microenvironment of the individual. It reflects the genetic and physiological characteristics of a specific individual well. This feature gives it a massive advantage in precision medicine in the future. However, there are several challenges to achieve this goal. In addition to the further standardization and modularization of organ-on-a-chip mentioned above, the stem cell differentiation program must be further optimized according to the degree of individualization. Significant progress needs to be made in the automation software for adjusting the culture parameters of each chip, in which dynamic culture parameters of multiple types of cells from different tissues or organs must be included in order to capture the characteristics of each phenotype of complex diseases in different individuals [115]. If the design and control of chips can reach the nanometer level, they can even be connected to the human body to replace diseased organs in the future. For example, a bionic lung constructed through organ-on-a-chip and related principles can provide respiratory support to patients. Compared with the existing artificial extracorporeal oxygenation membrane, this bionic lung is more portable and has a larger total gas exchange capacity due to the elimination of gas cylinders and pumps and the direct integration of heaters and sensors into the equipment [116]. In the future, the development of organ-on-a-chip has the potential to improve clinical results and the quality of life of patients significantly.
